# Gender-specific association between the regular use of statins and the risk of irritable bowel syndrome: A population-based prospective cohort study

**DOI:** 10.3389/fphar.2022.1044542

**Published:** 2023-01-06

**Authors:** Xiaoying Zhang, Yuyao Liu, Yanghui Ou, Man Yang, Jinqiu Yuan, Qiangsheng He, Yanfei Li, Ningning Mi, Peng Xie, Wenjing Li, Siqin Wu, Xiwen Qin, Jian Qi, Bin Xia

**Affiliations:** ^1^ Health Management Center, The Seventh Affiliated Hospital, Sun Yat-sen University, Shenzhen, China; ^2^ School of Medicine, Sun Yat-sen University, Shenzhen, China; ^3^ Center for Digestive Disease, The Seventh Affiliated Hospital, Sun Yat-sen University, Shenzhen, China; ^4^ Clinical Research Center, The Seventh Affiliated Hospital, Sun Yat-sen University, Shenzhen, , China; ^5^ Big Data Center, The Seventh Affiliated Hospital, Sun Yat-sen University, Shenzhen, China; ^6^ Chinese Health Risk Management Collaboration (CHRIMAC), Shenzhen, China; ^7^ Evidence Based Medicine Center, School of Basic Medical Sciences, Lanzhou University, Lanzhou, China; ^8^ Special Minimally Invasive Surgery Department, The First Hospital of Lanzhou University, Lanzhou, China; ^9^ Department of Pharmacology and Pharmacy, LKS Faculty of Medicine, The University of Hong Kong, Hong Kong, Hong Kong SAR, China; ^10^ Laboratory of Data Discovery for Health (D24H), The University of Hong Kong, Hong Kong, Hong Kong SAR, China; ^11^ School of Population and Global Health, Faculty of Medicine, Density and Health Sciences, University of Western Australia, Perth, AU-WA, Australia

**Keywords:** irritable bowel syndrome, statins (3-hydroxy-3-methyl-glutaryl-CoA reductase inhibitors), cohort study [or longitudinal study], protective factor, United Kingdom Biobank

## Abstract

**Introduction:** In addition to lipid-lowering effects, statins might modulate the gut microbiome and alleviate systematic inflammation, which in turn, may have a protective effect against irritable bowel syndrome (IBS). The aim of our study was to evaluate the gender-specific association between statin exposure and the risk of IBS.

**Method:** We undertook a prospective analysis based on the United Kingdom Biobank, a large ongoing cohort including 477,293 participants aged 37–73 years. We included participants based on information on their personal statin use and also those free of IBS and cancer at the baseline. We evaluated the gender-specific hazard ratio (HR) and 95% confidence interval (CI) with Cox proportional hazards regression, adjusting for demographic factors, lifestyle factors, comorbidities, and statin indications.

**Result:** A total of 438,805 participants (206,499 males and 232,306 females) were included in the analysis. Among male participants, the regular use of statins was associated with a decreased risk of IBS (HR: 0.77; 95% CI: 0.61–0.97). This association persists across multiple sensitivity and subgroup analyses and did not show clear evidence of variance among the major types of statins. We did not find sufficient evidence of the association between the statin use and IBS risk in females (HR: 0.98; 95% CI: 0.82–1.16).

**Conclusion:** Our study found that the regular use of statins was associated with a decreased risk of IBS in male participants. Further studies are required to confirm the beneficial effect of statins.

## 1 Introduction

Irritable bowel syndrome (IBS) is a chronic symptom-based functional gastrointestinal disorder characterized by abdominal pain or discomfort and disturbed bowel action (Grayson, 2016). The prevalence of IBS varies greatly internationally, with pooled prevalence estimates ranging from 5% to 20% of the global population (Sperber et al., 2017). Although IBS has no attributable mortality, it is associated with psychological comorbidities and other extraintestinal somatic symptoms and has a major impact on the quality of life and work productivity (Chey et al., 2015). In addition to its impact on public health, studies also suggest that the economic burden of IBS is quite substantial, with direct and indirect costs for healthcare resource utilization of the disease ranging from £45.6–200 million per annum in the United Kingdom to $2 billion per annum in China and €3−4 billion per annum in Germany (Black and Ford, 2020).

The pathogenesis of IBS remains unsettled and, in all probability, is multifactorial and driven by the dysbiosis of the gut microbiome in a genetically susceptible host (Holtmann et al., 2016). Prior studies also suggested that the dysbiosis of the gut microbiome (Holtmann et al., 2016), enteric infection (Berumen et al., 2021), disorders of gut−brain interaction (DGBI) (Enck et al., 2016), gut inflammation, and mucosal immune activation might be involved in IBS pathogenesis (Barbara et al., 2021). Evidence from pharmacoepidemiology studies also shows that the exposure to proton pump inhibitors (PPIs), metformin, and antibiotics, which might disturb intestinal homeostasis by altering the composition and diversity of the gut microbiota, is associated with an increased incidence of IBS and its related symptoms (Bytzer et al., 2001; Keszthelyi et al., 2012; Lee et al., 2013).

Statins, the potent lipid-lowering agents for the primary and secondary prevention of cardiovascular diseases and treatment of dyslipidemia (Chou et al., 2016), are among the most widely prescribed medications globally (Desai et al., 2014). In addition to the efficacy of lipid-lowering agents, statins can also downregulate systematic inflammation (Sorensen et al., 2019) and facilitate the gut bacteria alteration into an anti-inflammatory profile, characterized by increased proportions of *Akkermansia muciniphila* and *Faecalibacterium prausnitzii* (Khan et al., 2018). Mechanically, a regular statin use might reduce the risk of IBS, but population-based epidemiological evidence remains limited. Emerging studies have suggested that the regular use of statins might relieve constipation in patients with IBS by ameliorating gut microbiota dysbiosis and regulating the metabolic activity of the intestinal microbiota (Vieira-Silva et al., 2020) (17). A recent study has also reported the protective effect of statins against the new onset inflammatory bowel disease (Peppas et al., 2020) (Lochhead et al., 2021).

Interestingly, it should be noted that sex- and gender-associated differences existed both in statin use and IBS incidence at the population level. Due to the side effects or sociocultural differences, women eligible for statin therapy were less likely to be treated with any statin or guideline-recommended statin intensity therapy than men and were more prone to decline and discontinue the treatment (Nanna et al., 2019). Additionally, previous studies have also suggested that sex hormones (e.g., sex steroids) seemed to play important roles in the pathogenesis of IBS (So and Savidge, 2021). Indeed, there are sex- and gender-associated differences in the subtypes and prevalence of IBS, with them being two to four times higher in women than they are in men, including the effectiveness of treatment for IBS (van Kessel et al., 2021). Given the sexual dimorphism in statin use and global disease burden of IBS, the investigation of their association might have major implications for gender-specific strategies for IBS prevention interventions and statin applications. To address this issue, we performed this prospective analysis based on the United Kingdom Biobank, a large ongoing cohort, to evaluate the association of statin exposure with the new onset of IBS separately in the male and female population.

## 2 Methods

### 2.1 Study design

This is a prospective cohort study based on the United Kingdom Biobank (application number 51671, approved August 2019). Between 2006 and 2010, 502,527 participants (229,131 males and 273,396 females) aged 37–73 years were recruited throughout England, Wales, and Scotland. A wide range of data, including the lifestyle, environment, medical history, and physical measures along with biological samples, were collected *via* a questionnaire and face-to-face interview at the baseline. A detailed description of the study design and survey methods of the United Kingdom Biobank cohort has been reported previously (Sudlow et al., 2015). In the present analysis, we included participants based on the information on their personal statin use and excluded those with a diagnosis of malignancy, IBS, inflammatory bowel disease, hematochezia, coeliac disease, and major psychotic episodes such as schizophrenia prior to the baseline. The United Kingdom Biobank has been approved by the North West Multi-centre Research Ethics Committee, the England and Wales Patient Information Advisory Group, and the Scottish Community Health Index Advisory Group. Written informed consent has been obtained from all participants.

### 2.2 Assessment of statin use

The self-reported regular use (defined as most days of the week for the last 4 weeks) of statin and its types (simvastatin, atorvastatin, and other statins) was first assessed by participants using a touchscreen questionnaire at the baseline at the United Kingdom Biobank assessment center, and it was then verified during verbal interviews with a research nurse. This assessment of the self-reported statin use was validated by a statin prescription, as previously reported, and was also used to define the regular statin use in other studies (Tran et al., 2020; Carter et al., 2022).

### 2.3 Assessment of main outcomes

Participants with IBS (ICD-10 code K58) were identified through a linkage to the hospital inpatient database, primary care database, and death registries (England and Wales: the Health and Social Care Information Center; Scotland: the National Health Service Central Register). Person-years was contributed by eligible participants from the recruitment date until the date of the first IBS diagnosis, death, or the end of the follow-up (28 February 2018), whichever occurred first.

### 2.4 Assessment of covariates

Data on the sociodemographic characteristics (i.e., age, sex, ethnicity, and the index of multiple deprivation), lifestyle habits (i.e., smoking, alcohol consumption, dietary intake, physical activity, and sleep duration), comorbidities (i.e., hypertension, diabetes mellitus, hypercholesterolemia, and cardiovascular diseases), and medications (multivitamins, mineral supplements, non-steroidal anti-inflammatory drugs [NSAIDs], aspirin, proton pump inhibitors [PPIs], beta-blockers, thiazide diuretics, angiotensin-converting enzyme inhibitors [ACEIs], calcium channel blockers, angiotensin II receptor blockers [ARBs], beta-blockers, and antipsychotics) were determined from the patient interview and touchscreen questionnaire at the baseline. The United Kingdom Biobank also collected data on the family history of IBS, overall health rating and the presence of longstanding illness, and disability or infirmity at the baseline. The body mass index (BMI, kg/m^2^) was calculated from the height and weight measured by trained research staff at the baseline. Physical activity was assessed with the International Physical Activity Questionnaire-Short Form (IPAQ-SF), and diet intake was assessed using the food frequency questionnaire, which has been validated in a previous study (Cassidy et al., 2016).

### 2.5 Statistical analysis

The distribution of the demographic and lifestyle characteristics of the study population according to the regular statin usage or gender was evaluated in descriptive analyses using Pearson’s χ^2^ test or *t*-test. The United Kingdom Biobank cohort was analyzed using the Cox regression with age as the underlying timescale to calculate gender-specific hazard ratios (HRs) and 95% CIs for statin use and IBS risk. For covariates with selections of “do not know” and “prefer not to answer,” or with missing covariate data, an “unknown/missing” response category was created. To address potential reverse causation, we lagged the exposure for 2 years, which could strengthen the temporality and allow a time window for IBS risk development.

We stratified the analyses jointly by age, United Kingdom Biobank assessment centers, and hypercholesterolemia status in the basic model. To control the possible confounding effects from sociodemographic characteristics and lifestyle factors, we additionally adjusted for race (*white* or *other*), BMI (*<18.5, 18.5–24.9, 25–30,* and *≥30*), menopausal status (for females only), index of multiple deprivation (a measure of socio-economic status), smoking status (*never smoked, previous smoker,* and *current smoker*), alcohol consumption (*never or special occasions only*, *one to three times a month*, *one to four times a week*, *daily*, *or almost daily*), physical activity (*MET hours/week*), sleep duration (*MET hours/day*), and portions of fruit and vegetable intake (*< 5 portions per day, ≥ 5 portions per day*, or *unknown/missing*) in the multivariable adjusted model 1. In the fully adjusted model 2, we additionally controlled for family history of IBS (*yes* or *no*), comorbidities (i.e., hypertension, diabetes mellitus, and cardiovascular diseases), and commonly used medications (including multivitamins, mineral supplements, NSAIDs, aspirin, PPIs, and hormone replacement therapy (for females only)). Considering the individual drug-specific effect, we also conducted the analysis by the type of statins (simvastatin, atorvastatin, and other statins). To present the association in a clinically useful way, we calculated number needed to treat (NNT) based on the fully-adjusted HR and incidence rate to estimate of the reduced risk caused by statin, with the method described by Altman and Andersen (1999) as following formula: NNT = 1/ARR. Where ARR = CER (Control Event Rate) − EER (Experimental Event Rate). To investigate potential effect modifiers, we conducted a subgroup analysis based on the fully-adjusted HR, according to age, BMI, smoking status, alcohol intake, physical activity, fruits and vegetable intake, the presence of mental and behavioural disorders, dyslipidemia or CVD, and menopausal status (for the females only). We calculated the interactions between subgroups, a *p* value <.05 was considered as statistically significant.

A number of sensitivity analyses were conducted to confirm the robustness of the primary results. First, we lagged the exposure for 4 years to avoid potential reverse causation. Second, due to potential drug interactions and management of IBS symptoms with other medications, we further controlled for other medications, such as thiazide diuretics, ACEIs, calcium channel blockers, ARBs, beta-blockers, and antipsychotics. Third, to minimize the potential bias from healthcare utilization, we adjusted the colonoscopy/sigmoidoscopy examination at the baseline. Fourth, we further controlled for the presence of some baseline comorbidity history (e.g., generalized anxiety disorder, neurotic depression, and sleep disorder) for their potential influence. Fifth, considering the potential bias from the overall health status, we adjusted the self-reported overall health rating (poor, fair, good, and excellent) and longstanding diseases (yes or no) at the baseline. At last, we performed a propensity-score matching analysis in the United Kingdom Biobank to test the robustness of the results. We performed all analyses by R software (version 3.5.0, R Foundation for Statistical Computing, Vienna, Austria).

## 3 Results

This study included a total of 438,805 participants (206,499 males and 232,306 females) in the United Kingdom Biobank, of which 44,183 male participants and 26,629 female participants were regular statin users. The baseline characteristics of included participants are presented in Table 1. Participants presented differences in terms of the demographic characteristics by gender, but male and female regular statin users had similar baseline characteristics. Compared with non-regular statin users, regular statin users were more likely to be older, obese, smokers, drinkers, and less physically active. As expected, they had higher rates of hypertension, hypercholesterolemia and other cardiovascular comorbidities (e.g., MI, CHD, CHF, and PVD), poor health rating, and longstanding illness.

**TABLE 1:** Baseline characteristics of statin use in the United Kingdom Biobank.

Characteristic	Male			Female		
	Non-regular statin user (N = 162,316)	Regular statin user (N = 44,183)	Overall (N = 206,499)	Non-regular statin user (N = 205,677)	Regular statin user (N = 26,629)	Overall (N = 232,306)
Mean (range) age, years	55.8 (37.4, 73.7)	61.6 (40.4, 70.3)^a^	57.0 (37.4, 73.7)	55.9 (39.7, 71.1)	62.0 (40.4, 70.3)^a^	56.6 (39.7, 71.1) &
White, N (%)	153423 (94.5)	41635 (94.2)	195058 (94.5)	194248 (94.4)	24816 (93.2)	219064 (94.3)^c^
Menopause, N (%)	N/A	N/A	N/A	137838 (67.0)	24547 (92.2)	162385 (69.9)
Mean (SD) index of multiple deprivation	17.3 (14.1)	18.9 (15.0)^a^	17.6 (14.3)	16.6 (13.5)	20.0 (15.4)^a^	17.0 (13.8) &
Mean (SD) BMI, kg/m^2^	27.5 (4.06)	29.3 (4.51)^a^	27.8 (4.22)	26.8 (5.01)	29.5 (5.65)^a^	27.1 (5.16) &
Never smoker, N (%)	85296 (52.5)	17300 (39.2)^a^	102596 (49.7)	125623 (61.1)	14661 (55.1)^a^	140284 (60.4) &
Never drinker, N (%)	20536 (12.7)	7 068 (16.0)^a^	27604 (13.4)	46591 (22.7)	9299 (34.9)^a^	55890 (24.1) &
Median (IQR) physical activity, MET hours/week	48.5 (50.5)	41.5 (44.4)^a^	47.0 (49.3)	42.4 (41.3)	39.6 (40.7)^a^	42.1 (41.2) &
>5 portions of fruits and vegetables per day, N (%)	49558 (30.5)	14920 (33.8)^a^	64478 (31.2)	88440 (43.0)	12319 (46.3)^a^	100759 (43.4) &
Hypertension, N (%)	98161 (60.5)	37958 (85.9)^a^	136119 (65.9)	14850 (7.2)	1541 (5.8)^a^	16391 (7.1) &
Hypercholesterolemia, N (%)	6519 (4.0)	42909 (97.1)^a^	49428 (23.9)	5448 (2.6)	26106 (98.0)^a^	31554 (13.6) &
Diabetes, N (%)	5282 (3.3)	11250 (25.5)^a^	16532 (8.0)	3434 (1.7)	5914 (22.2)^a^	9348 (4.0) &
Stroke	1242 (0.8)	2776 (6.3)^a^	4018 (1.9)	1184 (0.6)	1484 (5.6)^a^	2668 (1.1) &
MI, N (%)	2016 (1.2)	11041 (25.0)^a^	13057 (6.3)	1062 (0.5)	2737 (10.3)^a^	3799 (1.6) &
CHD, N (%)	3062 (1.9)	13101 (29.7)^a^	16163 (7.8)	2379 (1.2)	4313 (16.2)^a^	6692 (2.9) &
CHF, N (%)	176 (0.1)	331 (0.7)^a^	507 (0.2)	89 (0.0)	112 (0.4)^a^	201 (0.1) &
PVD, N (%)	900 (0.6)	1243 (2.8)^a^	2143 (1.0)	1786 (0.9)	532 (2.0)^a^	2318 (1.0)
Multivitamin use, N (%)	19238 (11.9)	5580 (12.6)^a^	24818 (12.0)	34750 (16.9)	4396 (16.5)^a^	39146 (16.9) &
Intake of mineral supplements, N (%)	29783 (18.3)	6 932 (15.7)^a^	36715 (17.8)	50140 (24.4)	5572 (20.9)^a^	55712 (24.0) &
Aspirin use, N (%)	15010 (9.2)	24512 (55.5)^a^	39522 (19.1)	12833 (6.2)	10589 (39.8)^a^	23422 (10.1) &
Non-aspirin NSAID use, N (%)	22975 (14.2)	4267 (9.7)^a^	27242 (13.2)	40140 (19.5)	3624 (13.6)^a^	43764 (18.8) &
PPI use, N (%)	11012 (6.8)	7813 (17.7)^a^	18825 (9.1)	15306 (7.4)	5658 (21.2)^a^	20964 (9.0)
ACEI use, N (%)	10061 (6.2)	17135 (38.8)^a^	27196 (13.2)	8751 (4.3)	7112 (26.7)^a^	15863 (6.8) &
ARB use, N (%)	2530 (1.6)	3729 (8.4)^a^	6259 (3.0)	3127 (1.5)	2411 (9.1)^a^	5538 (2.4) &
Beta-blocker use, N (%)	4962 (3.1)	11212 (25.4)^a^	16174 (7.8)	6490 (3.2)	4829 (18.1)^a^	11319 (4.9) &
Poor health rating, N (%)	5259 (3.2)	4218 (9.5)^a^	9477 (4.6)	5467 (2.7)	2128 (8.0)^a^	7 595 (3.3) &
Longstanding illness, N (%)	41958 (25.8)	24937 (56.4)^a^	66895 (32.4)	47952 (23.3)	13452 (50.5)^a^	61404 (26.4) &

^a^ Presents *p*-value < 0.001 of the distribution difference of the demographic characteristics of the study population according to the regular statin status (yes/no) evaluated in descriptive analyses using the Pearson’s χ.

^b^ Test or *t*-test.

^c^ Presents *p*-value < 0.05 and ^&^ present *p*-value < 0.001 of the distribution difference of the demographic characteristics of the study population according to the gender evaluated in descriptive analyses using Pearson’s χ^2^ test or *t*-test.

Abbreviations: BMI, body mass index; MET, metabolic equivalent task; MI, myocardial infarction; CHD, coronary heart disease; CHF, congestive heart failure; PVD, peripheral vascular disease; NSAID, non-steroidal anti-inflammatory drug; PPI, proton pump inhibitor; ACEI, angiotensin-converting enzyme inhibitor; ARB, angiotensin receptor inhibitor.

During the 4,891,600 person-years of follow-up in the United Kingdom Biobank, we identified 1,846 incident cases of IBS among male participants (statin user: n = 386; non-statin user: n = 1,460) and 4,393 incident cases of IBS among female participants (statin user: n = 663; non-statin user: n = 3,370), respectively. Table 2 presents the gender-specific association between the regular statin use and the risk of IBS. In the crude model, statin use was associated with a 23% lower risk of IBS among male participants (HR: 0.77; 95% CI: 0.61–0.97; Table 2). We did not find sufficient evidence of the associations between the regular statin use and the risk of IBS among female participants (HR: 0.98; 95% CI: 0.82–1.16; Table 2). These results presented consistently across all the multi-adjusted models among male participants (adjust HR: 0.76; 95% CI: 0.6–0.96 and adjust HR: 0.73; 95% CI: 0.58–0.92, respectively; Table 2) and female participants (adjust HR: 0.92; 95% CI: 0.77–1.10 and adjust HR: 0.87; 95% CI: 0.73–1.04, respectively; Table 2) after adjusting for demographic factors, lifestyle habits, the presence of comorbidities, and the use of other medications. For easy interpretation of the effect, we calculated NNT based on the fully adjusted HR and incidence rate. Every 213 (95% CI, 66–1618) regular statin user can prevent one case of IBS over a median follow-up of 11.4 years.

**TABLE 2:** Hazard ratios (95% confidence intervals) for the association between regular statin use and the risk of inflammatory bowel disease.

	Male		Female	
	Non-regular statin user	Regular statin user	Non-regular statin user	Regular statin user
Number of events	1460	386	3730	663
Person-years	1814694	478823	2305583	292500
HR (95% CI)				
Crude model^a^	1.00 (Ref)	0.77 (0.61, 0.97)	1.00 (Ref)	0.98 (0.82, 1.16)
Multivariate-adjusted model 1^b^	1.00 (Ref)	0.76 (0.6, 0.96)	1.00 (Ref)	0.92 (0.77, 1.1)
Multivariate-adjusted model 2^c^	1.00 (Ref)	0.73 (0.58, 0.92)	1.00 (Ref)	0.87 (0.73, 1.04)

^a^ Crude model: basic Cox proportional hazards model stratified by age, United Kingdom Biobank assessment centers, and hypercholesterolemia status.

^b^ Multivariate-adjusted model 1: additionally adjusted for race (*white* or *other*), BMI (*<18.5, 18.5–24.9, 25–30,* and *≥30*), menopausal status (for females only), index of multiple deprivation (a measure of socio-economic status), smoking status (*never smoked, previous smoker,* and *current smoker*), alcohol consumption (*never or special occasions only*, *one to three times a month*, *one to four times a week*, *daily*, *or almost daily*), physical activity (*MET, hours/week*), sleep duration (*MET, hours/day*), portions of fruit and vegetable intake (*< 5 portions per day, ≥ 5 portions per day*, or *unknown/missing*).

^c^ Multivariate-adjusted model 2: fully adjusted model additionally adjusted for IBS (*yes* or *no*), comorbidities (i.e., hypertension, diabetes mellitus, and cardiovascular diseases), and commonly used medications (including multivitamins, mineral supplements, NSAIDs, aspirin, PPIs, and hormone replacement therapy (for females only)).

**FIGURE 1:**
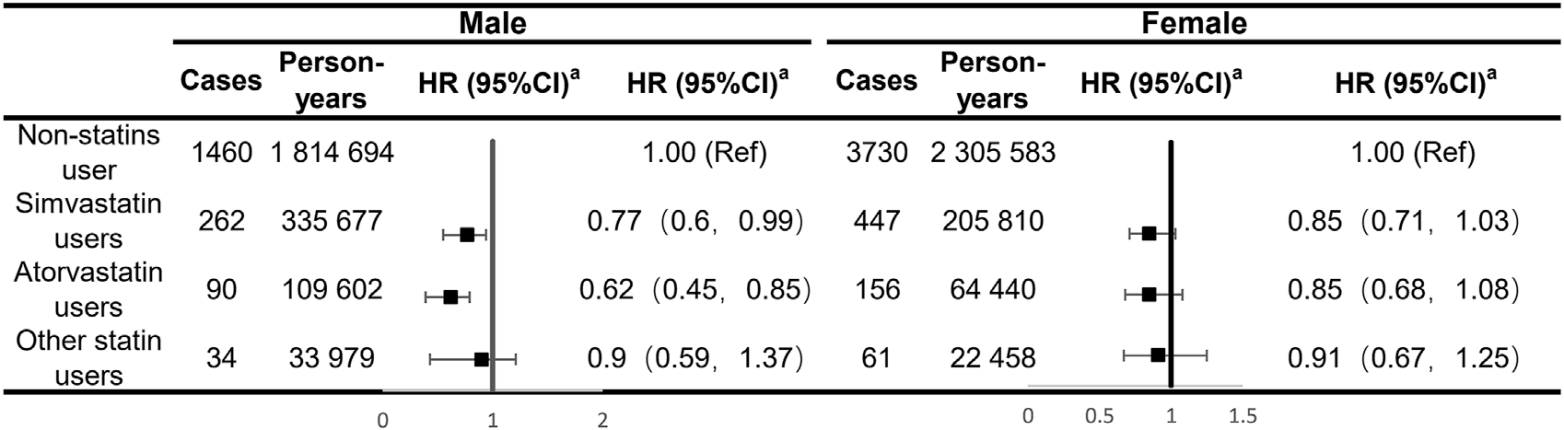
Analyses of the regular use of statins and risk of irritable bowel syndrome adjusting for major types of statins. ^a^Estimated effects were based on the multivariable adjusted Cox proportional hazards model additionally adjusted for race (white or other), BMI (<18.5, 18.5–24.9, 25–30, and ≥30), menopausal status (for females only), index of multiple deprivation (a measure of socio-economic status), smoking status (never smoked, previous smoker, and current smoker), alcohol consumption (never or special occasions only, one to three times a month, one to four times a week, daily, or almost daily), physical activity (MET hours/week), sleep duration (MET hours/day), portions of fruit and vegetable intake (<5 portions per day, ≥5 portions per day, or unknown/missing), IBS (yes or no), comorbidities (i.e., hypertension, diabetes mellitus, and cardiovascular diseases), and commonly used medications (including multivitamins, mineral supplements, NSAIDs, aspirin, PPIs, and hormone replacement therapy (for females only)).

**FIGURE 2:**
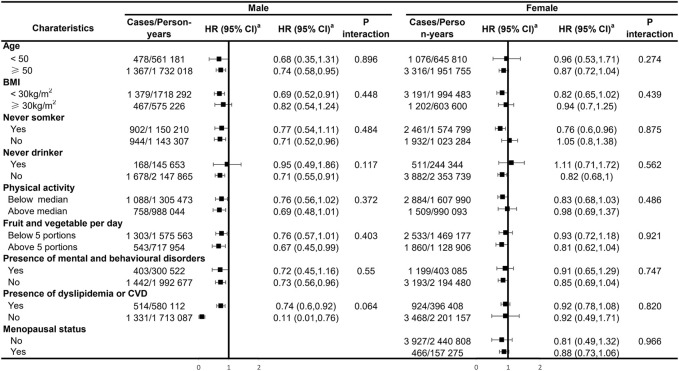
Subgroup analyses of the regular use of statins and risk of irritable bowel syndrome. ^a^Estimated effects were based on the multivariable adjusted Cox proportional hazards model additionally adjusted for race (white or other), BMI (<18.5, 18.5–24.9, 25–30, and ≥30), menopausal status (for females only), index of multiple deprivation (a measure of socio-economic status), smoking status (never smoked, previous smoker, and current smoker), alcohol consumption (never or special occasions only, one to three times a month, one to four times a week, daily, or almost daily), physical activity (MET hours/week), sleep duration (MET hours/day), portions of fruit and vegetable intake (<5 portions per day, ≥5 portions per day, or unknown/missing), IBS (yes or no), comorbidities (i.e., hypertension, diabetes mellitus, and cardiovascular diseases), and commonly used medications (including multivitamins, mineral supplements, NSAIDs, aspirin, PPIs, and hormone replacement therapy (for females only)).

Our analyses for the individual class of statins generally showed a negative association with the risk of IBS in males, with significant effects observed for simvastatin and atorvastatin (HR: 0.77; 95% CI: 0.60–0.99 and HR: 0.62; 95% CI: 0.45–0.85, respectively; Figure 1). There was no sufficient evidence of the association between each type of statins and the risk of IBS among female participants. In subgroup analyses, statin-associated preventive effects of IBS did not differ by age, obesity, smoking and drinking status, physical activity, dietary quality, the presence of mental and behavioral disorders, and dyslipidemia or CVD status in males, suggesting there was no interaction with any of these factors (Figure 2). For female participants, the observed associations were generally similar across cohort subgroups, which did not achieve statistical significance.

The primary results were robust across sensitivity analyses (Table 3). We observed no major changes in the association between statin and IBS after lagging the exposure for 4 years (HR: 0.75; 95% CI: 0.57–0.99 for males and HR: 0.84; 95% CI: 0.69–1.03 for females, respectively), additionally adjusting for other commonly used drugs (HR: 0.72; 95% CI: 0.57–0.91 for males and HR: 0.87; 95% CI: 0.73–1.04 for females, respectively). We additionally adjusted for the colonoscopy/sigmoidoscopy examination (HR: 0.72; 95% CI: 0.57–0.92 for males and HR: 0.88; 95% CI: 0.74–1.05 for females, respectively), additionally adjusting for the presence of IBS-related neurological disorders at the baseline (HR: 0.72; 95% CI: 0.57–0.92 for males and HR: 0.88; 95% CI: 0.74–1.05 for females, respectively), and we additionally adjusted the self-reported overall health rating and longstanding diseases at the baseline (HR: 0.72; 95% CI: 0.57–0.92 for males and HR: 0.88; 95% CI: 0.74–1.05 for females, respectively). After propensity-score matching of 1:4, statin use was also associated with a decreased risk of IBS (HR: 0.66; 95% CI: 0.47–0.91 for males and HR: 0.98; 95% CI: 0.80–1.22 for females, respectively) (Table 3).

**TABLE 3:** Hazard ratios (95% confidence intervals) for associations between regular statin use and the risk of irritable bowel syndrome in sensitivity analyses.

	Male		Female	
	Non-regular statin user	Regular statin user	Non-regular statin user	Regular statin user
Multivariate-adjusted HR (95% CI)^a^	1.00 (Ref)	0.75 (0.57, 0.99)	1.00 (Ref)	0.84 (0.69, 1.03)
Multivariate-adjusted HR (95% CI)^b^	1.00 (Ref)	0.72 (0.57, 0.91)	1.00 (Ref)	0.87 (0.73, 1.04)
Multivariate-adjusted HR (95% CI)^c^	1.00 (Ref)	0.72 (0.57, 0.92)	1.00 (Ref)	0.88 (0.74, 1.05)
Multivariate-adjusted HR (95% CI)^d^	1.00 (Ref)	0.72 (0.57, 0.92)	1.00 (Ref)	0.88 (0.74, 1.05)
Multivariate-adjusted HR (95% CI)^e^	1.00 (Ref)	0.72 (0.57, 0.92)	1.00 (Ref)	0.88 (0.74, 1.05)
Multivariate-adjusted HR (95% CI)^f^	1.00 (Ref)	0.66 (0.47, 0.91)	1.00 (Ref)	0.98 (0.8, 1.22)

^a^ Estimated effects were based on the fully adjusted model (see the footnote in Table 2) after lagging the exposure for 4 years.

^b^ Estimated effects were based on the fully adjusted model (see the footnote in Table 2) and additionally adjusted for other medications, such as beta-blockers, thiazide diuretics, ACEIs, calcium channel blockers, ARBs, beta-blockers, and antipsychotics.

^c^ Estimated effects were based on the fully adjusted model (see the footnote in Table 2) and additionally adjusted for the colonoscopy/sigmoidoscopy examination at the baseline.

^d^ Estimated effects were based on the fully adjusted model (see the footnote in Table 2) and additionally adjusted for the presence of psychiatric comorbidities (generalized anxiety disorder, neurotic depression, and sleep disorder).

^e^ Estimated effects were based on the fully adjusted model (see the footnote in Table 2) and additionally adjusted for participants with hypercholesterolemia.

^f^ Estimated effects were based on the propensity score method.

## 4 Discussion

In this prospective cohort study based on the United Kingdom Biobank, we found that among male participants, the regular use of statin was associated with a 23% decreased risk of IBS, while we did not find sufficient evidence for the association between statin use and the reduced risk of IBS among female participants. This result persisted across multiple sensitivity and subgroup analyses and did not show clear evidence of variance among the major types of statins.

To the best of our knowledge, few studies have directly evaluated the statin use and IBS risk. Emerging evidence has found that lovastatin lactone might improve irritable bowel syndrome with constipation (IBS-C) by inhibiting enzymes in the archaeal methanogenesis pathway (Muskal et al., 2016). This study, at least partly, supported our findings that subjects with regular use of statin were associated with a lower risk of IBS than those without statin regular usage. Previous studies have also revealed the association between statin use and the reduced risk of IBS-related symptoms. For instance, a propensity score-matched cohort study of 12,684 patients found that regular statin users had a lower prevalence of diarrhea and colitis, and statin use was associated with a decreased risk of diarrhea (Pearlman et al., 2017; Suchartlikitwong et al., 2018). In a randomized controlled phase 2a trial, a proprietary modified-release formulation of lovastatin lactone lowered breath methane levels and improved stool frequency in patients with IBS-C (Gottlieb et al., 2016), which further suggested the potential protective effects of statins on IBS development.

There are several potential mechanisms regarding the protective role of statins on IBS development. Prior studies indicated that gut microbiota dysbiosis is a pathogenetic and pathophysiological factor of IBS (Mars et al., 2020). Statins may modulate the gut microbiome by altering cholesterol metabolism and the bile acid profile (Li et al., 2022). This may reduce the intestinal flora *Bacteroides* 2 enterotype (Ghoshal et al., 2016), a gut microbiota configuration that is highly prevalent in people with IBS (Duan et al., 2019; Pittayanon et al., 2019). Individuals who received statin therapy also presented a higher level of probiotics, such as *Lactobacillus* and *Bifidobacterium* genera (Dias et al., 2020), which were found in lower amounts in patients with IBS (27) (Zhuang et al., 2017). Moreover, statins have an anti-methanogenesis effect through the direct inhibition of enzymes in the archaeal methanogenesis pathway (17), which is critical in aggravating constipation in IBS (Ghoshal et al., 2016). As gastrointestinal infection and inflammation are commonly identified risk factors for the development of IBS (Berumen et al., 2021), statins might play an immunomodulatory and anti-inflammatory function by attenuating pro-inflammatory cytokine release and anti-oxidant effects (Nakagomi et al., 2012), which might be beneficial for IBS development.

In the present study, regular statin use was associated with a decreased risk of IBS in males only. The potential mechanisms underlying the gender discrepancy remained unclear but might be, at least partly, related to the sexual dimorphism in pharmacokinetics of the statin metabolism. Males expressed less than half as much CYP3A4 (a major hepatic cytochrome P-450 that metabolizes all lipophilic statins) than women. This might lead to a slower and less extensive statin metabolism in males than females; hence, the statin pharmaceutical activity was correspondingly higher (Raparelli et al., 2017). Furthermore, the disparities between men and women in medication adherence may also influence the potential efficacy of statin in IBS prevention. Indeed, men were reported less likely to switch or stop a statin because of worrying about side effects than women (Karalis et al., 2016). On the other hand, it is speculated that statins might not entirely offset the increased risk of IBS related to estrogen in women. Estrogen might delay intestinal motility (Fischer and Fadda, 2016), possibly through the modulation of the nitric oxide (NO)/cyclic guanosine monophosphate (cGMP) pathway (Jung et al., 2003). A slower intestinal transit in women than in men contributed to the predominance of IBS with constipation (IBS-C) (34). Furthermore, by activating mast cell degranulation (Mackey et al., 2016), estrogen enhanced histamine and cytokine release (Zaitsu et al., 2007), inducing visceral hypersensitivity and increasing the intestinal permeability (Kleij and Bienenstock, 2005; Uranga et al., 2020). Female IBS patients also showed greater 5-HT synthesis in the brain than IBS males (Nishizawa et al., 1997), which might evoke enteric nervous system responses (Kim and Camilleri, 2000) and enhance smooth muscle contraction to cause pain and bloating associated with IBS (Stasi et al., 2019). Further studies are needed to confirm our findings and investigate the potential mechanisms underlying the gender discrepancy of the statin-associated preventive effects on IBS.

A key strength in the present study is that our data were based on a nationwide prospective United Kingdom Biobank database, with detailed measurements and long-term follow-up durations. We evaluated the association between statin use and IBS risk by gender, which was seldom reported in previous epidemiological studies. The availability of a wide range of known and putative risk factor data from the United Kingdom Biobank allowed for simultaneous adjustability of potential confounders for the association of interests. The results’ consistency from several robust sensitivity analyses also increased our confidence in these findings.

Limitations also exist in our study. First, despite the comprehensive adjustment for confounders, residual confounding effects could not be ruled out completely. Second, there was a chance of misclassification of statin use during the follow-up in the United Kingdom Biobank because statin use was only evaluated once at the baseline. However, misclassification would underestimate the true effect because the control group included many statin users with a lower IBS risk. So, the potential misclassification would be unlikely to change our main findings. Third, the definition of regular statin use in the present study was not precise enough as information about the statin use dosage, frequency, and duration was not collected in the United Kingdom Biobank, and the data about the midway alteration of statin types were lacking. This might limit our ability to comprehensively assess the relationship between the regular statin use and IBS risk. Further prospective studies with detailed medication exposures are needed to validate our findings. Finally, despite sensitivity analyses introducing a 4-year lag, we cannot fully eliminate reverse causation. However, the influence is minor because according to the Rome Ⅲ/Ⅳ criteria, the shortest time from the onset of symptoms to a diagnosis of IBS is 6 months (Drossman and Hasler, 2016), and most likely, an even longer lagging period yielded similar findings.

## Conclusion

Our findings from this large, prospective study of British adults showed that the regular use of statin was associated with a decreased risk of IBS among males, regardless of major types of statins. Our study provided clues for hypothesis generation and might extend the potential benefit of regular statin use in digestive health. However, this association should be interpreted with caution as the study design is observational. Further studies, particularly well-designed prospective cohort studies, are needed to confirm this beneficial effect of statins. Investigation of the potential mechanisms behind this association is also necessary, which may lead to preventive strategies for patients at a higher risk of IBS.

## Data Availability

The original contributions presented in the study are included in the article/Supplementary Materials; further inquiries can be directed to the corresponding authors.
